# Mortality rates of severe COVID-19-related respiratory failure with and without extracorporeal membrane oxygenation in the Middle Ruhr Region of Germany

**DOI:** 10.1038/s41598-023-31944-7

**Published:** 2023-03-29

**Authors:** Assem Aweimer, Lea Petschulat, Birger Jettkant, Roland Köditz, Johannes Finkeldei, Johannes W. Dietrich, Thomas Breuer, Christian Draese, Ulrich H. Frey, Tim Rahmel, Michael Adamzik, Dirk Buchwald, Dritan Useini, Thorsten Brechmann, Ingolf Hosbach, Jürgen Bünger, Aydan Ewers, Ibrahim El-Battrawy, Andreas Mügge

**Affiliations:** 1 Department of Cardiology and Angiology, BG University Hospital Bergmannsheil, Ruhr-Universität Bochum, Bürkle-de-La-Camp-Platz 1, 44789 Bochum, Germany; 2 Institute for Prevention and Occupational Medicine of the German Social Accident Insurance, Institute of the Ruhr-Universität Bochum (IPA), Bochum, Germany; 3 Department of Endocrinology and Diabetes, BG University Hospital Bergmannsheil, Ruhr University of Bochum, Bochum, Germany; 4 Diabetes, Endocrinology and Metabolism Section, Medical Hospital I, Katholisches Klinikum Bochum, St Josef Hospital Bochum, Ruhr University Bochum, Bochum, Germany; 5 Department of Internal Medicine, Katholisches Klinikum Bochum, St. Josef-Hospital, Ruhr-University Bochum, Bochum, Germany; 6 Klinik für Anästhesiologie, Operative Intensivmedizin, Schmerz- und Palliativmedizin, Marien Hospital Herne, Universitätsklinikum der Ruhr-Universität Bochum, Bochum, Germany; 7 Klinik für Anästhesiologie, Intensivmedizin und Schmerztherapie, Universitätsklinikum Knappschaftskrankenhaus Bochum, Bochum, Germany; 8 Department of Cardiothoracic Surgery, BG University Hospital Bergmannsheil, Ruhr-University Bochum, Bochum, Germany; 9 Gastroenterology and Hepatology, BG University Hospital Bergmannsheil, Bochum, Germany

**Keywords:** Diseases, Viral infection

## Abstract

The use of extracorporeal membrane oxygenation (ECMO) is discussed to improve patients’ outcome in severe COVID-19 with respiratory failure, but data on ECMO remains controversial. The aim of the study was to determine the characteristics of patients under invasive mechanical ventilation (IMV) with or without veno-venous ECMO support and to evaluate outcome parameters. Ventilated patients with COVID-19 with and without additional ECMO support were analyzed in a retrospective multicenter study regarding clinical characteristics, respiratory and laboratory parameters in day-to-day follow-up. Recruitment of patients was conducted during the first three COVID-19 waves at four German university hospitals of the Ruhr University Bochum, located in the Middle Ruhr Region. From March 1, 2020 to August 31, 2021, the charts of 149 patients who were ventilated for COVID-19 infection, were included (63.8% male, median age 67 years). Fifty patients (33.6%) received additional ECMO support. On average, ECMO therapy was initiated 15.6 ± 9.4 days after symptom onset, 10.6 ± 7.1 days after hospital admission, and 4.8 ± 6.4 days after the start of IMV. Male sex and higher SOFA and RESP scores were observed significantly more often in the high-volume ECMO center. Pre-medication with antidepressants was more often detected in survivors (22.0% vs. 6.5%; *p* = 0.006). ECMO patients were 14 years younger and presented a lower rate of concomitant cardiovascular diseases (18.0% vs. 47.5%; *p* = 0.0004). Additionally, cytokine-adsorption (46.0% vs. 13.1%; *p* < 0.0001) and renal replacement therapy (76.0% vs. 43.4%; *p* = 0.0001) were carried out more frequently; in ECMO patients thrombocytes were transfused 12-fold more often related to more than fourfold higher bleeding complications. Undulating C-reactive protein (CRP) and massive increase in bilirubin levels (at terminal stage) could be observed in deceased ECMO patients. In-hospital mortality was high (Overall: 72.5%, ECMO: 80.0%, ns). Regardless of ECMO therapy half of the study population deceased within 30 days after hospital admission. Despite being younger and with less comorbidities ECMO therapy did not improve survival in severely ill COVID-19 patients. Undulating CRP levels, a massive increase of bilirubin level and a high use of cytokine-adsorption were associated with worse outcomes. In conclusion, ECMO support might be helpful in selected severe cases of COVID-19.

## Introduction

COVID-19 associated pneumonia, derived from SARS-Cov2 infection, led to high and critical occupancy of intensive care resources around the world and stressed the healthcare system capacities immensely. In a surveillance study in the United States 14% of COVID-19 patients were hospitalized, 2% were admitted to an intensive care unit (ICU), and 5% died^1^. The intra-hospital mortality of hospitalized patients summed up to 20% due to progression to COVID-19 related life-threatening complications, e.g. acute respiratory distress syndrome (ARDS), septic shock, or multiorgan failure requiring oxygen support or invasive mechanical ventilation (IMV)^2,3^. In case of insufficient oxygenation or decarboxylation despite IMV and prone positioning a veno-venous extracorporeal membrane oxygenation (ECMO) could become necessary. Supporting data derived from the successful management of severe respiratory failure in patients with H1N1 influenza A and Middle East respiratory syndrome^4,5^. Therefore, with growing numbers of severe ARDS during the COVID-19 pandemic the use of ECMO support increased depending on available resources.

Compared to ARDS of other etiology COVID-19 patients with ECMO support stay longer on ICU^6^. Increased length of ICU stay and mortality are worrying, especially in the pre-vaccination era. Nevertheless, unvaccinated people or breakthrough infections are real challenges for health care systems in the future. The COVID-19 pandemic continues to be severe, particularly in certain population groups. The mortality rates due to COVID-19 related ARDS ranges between 54 and 76%^7–10^.

Recent studies with severe ARDS in COVID-19 showed a beneficial effect of ECMO use with mortality rates of 30–60%^11–14^. Karagiannidis et al. reported a higher mortality rate of 71% during the first wave of the pandemic in German hospitals^15^. In a further analysis these data were confirmed with in-hospital mortality of 73% on average and more than 80% in patients older than 60 years, respectively^16^. However, these German studies are based on registry data and not attributable to a distinct region.

Recent data show that the mortality rates attributable to COVID 19 differ widely across countries or within regions in the same country^17–19^, so that regional differences could also affect intensive care conditions like need of IMV and ECMO support.

In the present study we analyzed retrospectively the clinical course of severe ARDS in COVID 19 at four German university hospitals of the Ruhr University Bochum, located in the Middle Ruhr Region (Germany’s largest urban area), during the first three COVID-19 waves. These university hospitals are all ARDS treatment centers with a cumulative capacity of 12 ECMO devices.

Hence, the purpose of this study was to summarize the characteristics and outcome parameters of these patients indicated for IMV due to ARDS with and without ECMO support to clarify its role and explore the high mortality rates in Germany.

## Methods

### Study design and participants

We performed a retrospective cohort study at four university hospitals of the Ruhr-University Bochum located in the Middle Ruhr Region of Germany. Consecutive adult (≥ 18 years) patients admitted to the ICU between March 1, 2020 and August 31, 2021, diagnosed with COVID-19 and supported by invasive mechanical ventilation were eligible for inclusion. Confirmation of severe acute respiratory syndrome coronavirus 2 (SARS-CoV-2) infection was based on a positive reverse transcriptase polymerase chain reaction (RT-PCR) assay (Cobas SARS-CoV-2 Test, Roche Molecular Systems, Branchburg, NJ, United States).

This non-interventional study was performed in agreement with the ethical principles and standards of the second Helsinki declaration and its later amendments. The study design was approved by the local institutional ethics committee of the Medical Faculty at the Ruhr University of Bochum, file number 21-7330-BR. In all the participating institutions, the requirement for patients informed consent was waived by ethics committee of the Medical Faculty at the Ruhr University of Bochum due to the retrospective nature of this study.

### Data collection

Data was collected from the patient data management system of all participating hospitals. The extracted data included demographics, comorbidities, Sequential Organ Failure Assessment (SOFA) at ICU admission, Charlson Comorbidity Index, resource use and organ support (vasopressors, noninvasive ventilation, prone positioning, IMV, veno-venous ECMO use, renal replacement therapy) during ICU stay, destination at hospital discharge, length of ICU and hospital stay, and ICU and in-hospital mortality, treatment modalities, transfusion of blood-derived products, particular medication such as glucocorticoid and anti-infective treatment, as well as laboratory test results in a day-by-day manner. Steroids used in all three waves were Dexamethasone, Hydrocortisone and Prednisolone, with Dexamethasone being used most often as first line steroid in all three waves.

Patients received veno-venous ECMO in case of refractory hypoxemia and/or hypercapnia despite ventilator optimization according to the ECMO to Rescue Lung Injury in Severe ARDS (EOLIA)’s criteria^18^. After enrollment, the patients were divided into ECMO and non-ECMO groups according to whether ECMO was applied. Surviving patients were followed up until hospital discharge.

### Statistical analysis

The collection and compilation of all patient data, treatment courses, diagnostic and laboratory values was retrieved from the respective patient files and collected in several spreadsheets. Laboratory-specific units, different calibrations and scales were uniformly converted. The merging of the various tables, the grouping as well as the automated graphical representation was carried out in Matlab Ver. 2020 (The MathWorks, Inc., Natick, MA 01760-2098, US). As a time index, the data contain both the days since admission to the hospital and days since the infection was detected (1st positive PCR test).

After the validity check a first descriptive statistical analysis of these raw data sets was done in Statistica Ver. 14, (TIBCO Software Inc. Palo Alto, CA 94304, US). Patient’s data were selected for a first statistical overview by ECMO treatment survival status. The various parameters of the patient groups were counted, averaged and finally compared using an unpaired t-test, a *p* value < 0.05 was considered significantly different. Patient characteristics are expressed as n (%) for categorical variables, mean (SD) for continuous variables, or median (IQR), as appropriate. The Survivor Functions for Two Groups were generated with the Matlab function ecdf() together with fitting Burr Type XII distributions and a regression of Cox proportional hazards model. The Poincaré plot as a special Recurrence plot was generated from raw data with Matlab’s plotting utilities^20^.

### Ethics approval and consent to participate

This non-interventional study was performed in agreement with the ethical principles and standards of the second Helsinki declaration and its later amendments. The study design was approved by the local institutional ethics committee of the Medical Faculty at the Ruhr University of Bochum, file number 21-7330-BR. In all the participating institutions, the requirement for patient consent was waived due to the retrospective nature of this study.

## Results

### Study population

**Figure 1:**
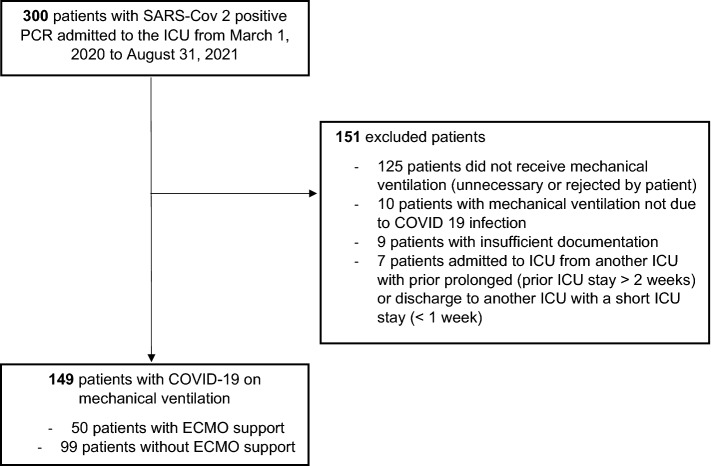
Enrollment flowchart ICU: intensive care unit, ECMO: extracorporeal membrane oxygenation.

**Figure 2:**
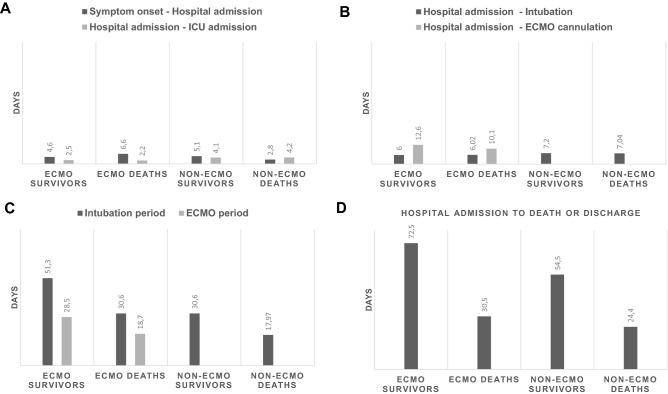
Days are shown as mean intervals for each group (ECMO survivors, ECMO deaths, Non-ECMO survivors, Non-ECMO deaths). (**A**) Intervals from symptom onset to hospital admission and hospital admission to ICU admission. (**B**) Intervals from hospital admission to intubation and hospital admission to ECMO cannulation. (**C**) Period of intubation (interval of intubation to extubation) and ECMO period (interval of ECMO cannulation to decannulation). (**D**) Intervals of hospital admission to death or discharge.

**Figure 3:**
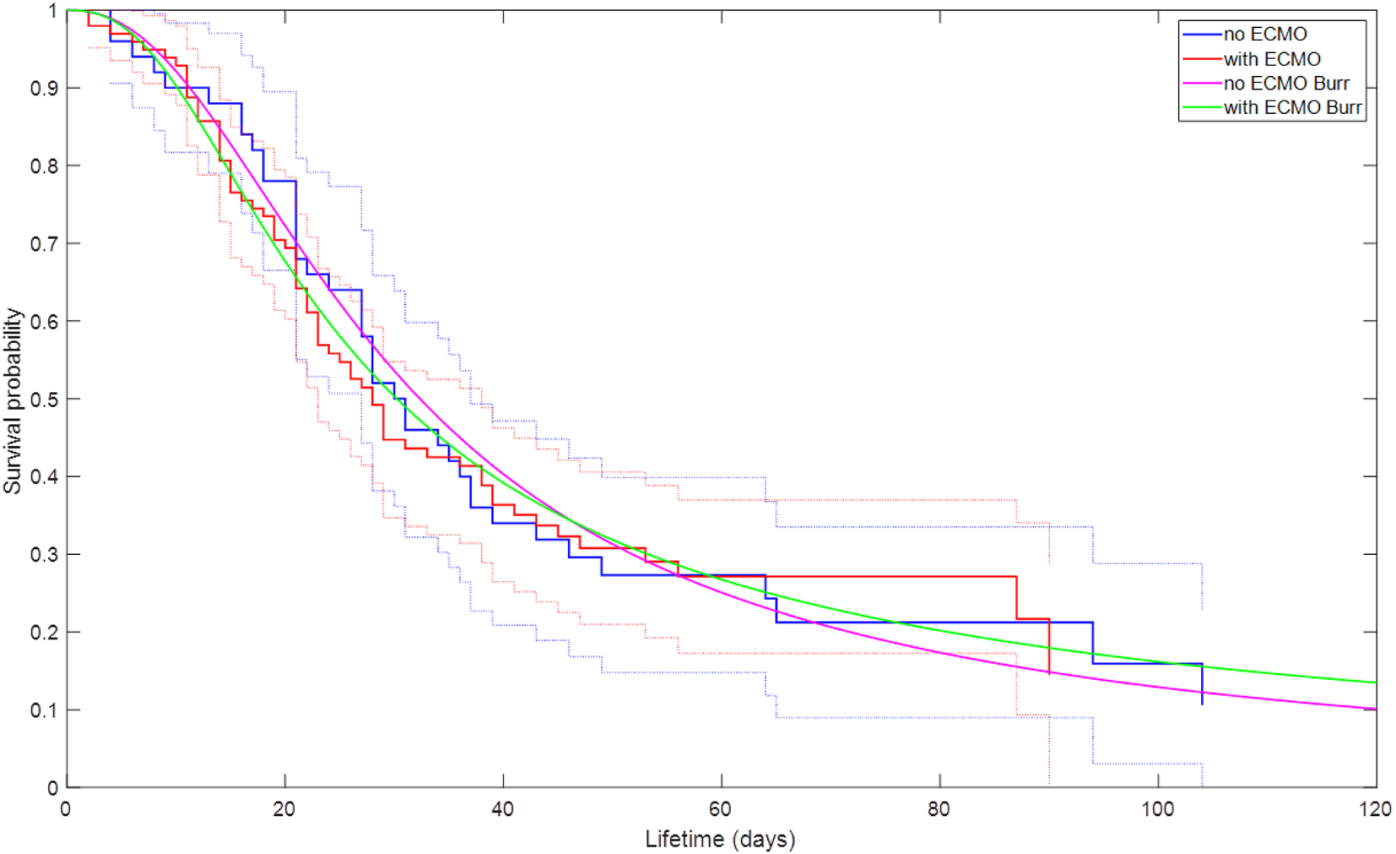
Kaplan Meier survival curve for hospital length, ECMO as explanatory variable and alive as censor variable with Burr fit and Cox proportional regression hazards model. *p* value = 0.91.

A total of 149 patients (63.8% male, median age 67 years, interquartile range: 60–76) were included who suffered from respiratory failure due to COVID-19 and were treated with IMV during ICU stay. Fifty patients (33.6%) received ECMO support (Fig. 1). As an exception, one patient was included to analysis who was at “awake’’ ECMO without need of IMV. On average, ECMO therapy was initiated 15.6 ± 9.4 days after symptom onset, 10.6 ± 7.1 days after hospital admission, and 4.8 ± 6.4 days after the start of IMV. Time periods dependent on symptom onset, hospital admission, ICU admission, intubation, ECMO initiation and death or discharge are summarized in Fig. 2. The longest period of symptom onset to hospital admission was observed for deceased ECMO patients (6.6 ± 7.4 days). Whereas surviving ECMO patients had the longest period from ICU admission to ECMO initiation (10.1 ± 9.0 days), the period from hospital admission to intubation was similar to deceased ECMO patients. IMV periods in surviving patients ranged from 30 to 50 days with particularly very long periods in ECMO patients (51.3 ± 18.0 days) compared to Non-ECMO patients (30.6 ± 18.9 days). Consecutively, the periods from admission to discharge were distributed similarly (ECMO: 72.5 ± 26.5 days, Non-ECMO 54.5 ± 25.0 days). The survival probability is shown in Fig. 3 as a Kaplan–Meier curve for ECMO and non-ECMO patients. No significant differences were observed between both groups (^p^ = 0.91). Half of the study population—independent of ECMO—deceased at 30 days after hospital admission.

### Characteristics of survivors and non-survivors

**Figure 4:**
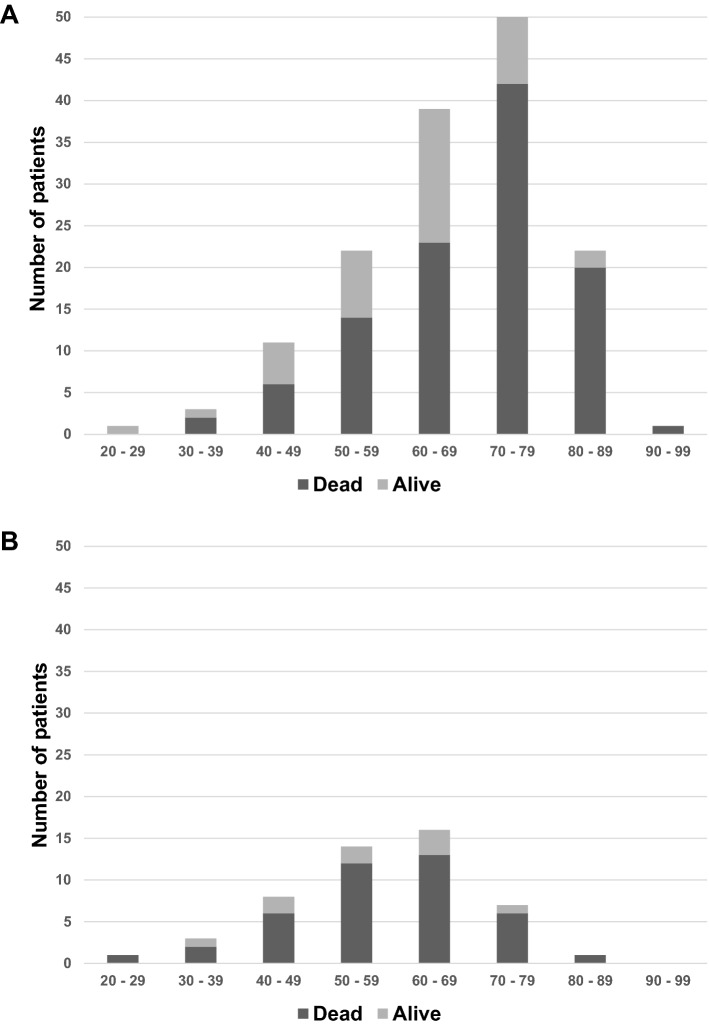
Survivors and deaths related to age groups are shown for the whole patient cohort (**A**) and especially for patients with ECMO support (**B**).

In Table 1 parameters are shown for survivors and non-survivors of the whole cohort. Overall mortality was summed up to 72.5%. Age structure dependent on survival status of the study population is shown in Fig. 4. The clinical characteristics between both differed only in a few parameters. For instance, non-survivors were older, got fewer tracheostomies, but more often corticosteroids during ICU treatment and cytokine adsorption therapy. Steroids used in all three waves were Dexamethasone, Hydrocortisone and Prednisolone, with Dexamethasone being used most often as first line steroid in all three waves. There were no significant differences in steroids used or duration of administration in the three waves. Concerning previous medication survivors took significantly more antidepressants and opioids and less alpha-antagonists compared to non-survivors (Supplementary Table 1).

**Table 1:** Basic characteristics of studied patients.

	All patients 149/149 (100%)	Non-survivors 108/149 (72.5%)	Survivors 41/149 (27.5%)	*p* Value
Age (median), years (min–max)	67 (24–90)	69 (36–90)	61 (24–82)	0.0004*
Male	95 (63.7%)	39 (63.9%)	26 (63.4%)	0.9575
ECMO-Therapy	50 (33.6%)	40 (37%)	10 (24.4%)	0.1462
Comorbidities	146 (98%)	106 (98.1%)	40 (97.6%)	0.8212
Hypertension	107 (71.8%)	77 (71.3%)	30 (73.2%)	0.8218
Dyslipidemia	49 (32.9%)	33 (30.6%)	16 (39.0%)	0.3291
Diabetes mellitus	54 (36.2%)	40 (37.0%)	14 (34.1%)	0.7451
Obesity	95 (63.8%)	69 (63.9%)	26 (63.4%)	0.9575
Current smoker	7 (4.7%)	2 (1.9%)	5 (12.2%)	0.0075*
Renal insufficiency	29 (19.5%)	20 (18.5%)	9 (22%)	0.6392
Allergies	29 (15.4%)	17 (15.7%)	6 (14.6%)	0.8685
Heart disease	56 (37.6%)	44 (40.7%)	12 (29.3%)	0.1991
Atrial fibrillation	26 (17.5%)	21 (19.4%)	5 (12.2%)	0.3010
Cerebrovascular disease	14 (9.4%)	12 (11.1%)	2 (4.9%)	0.2471
Autoimmun disease	1 (0.7%)	1 (0.9%)	0 (0.0%)	0.5396
Connective tissue disease	1 (0.7%)	1 (0.9%)	0 (0.0%)	0.5396
Liver disease	3 (2%)	2 (1.9%)	1 (2.4%)	0.8212
Any cancer	15 (10.1%)	13 (12.0%)	2 (4.9%)	0.1971
Immunsupression condition	4 (2.7%)	3 (2.8%)	1 (2.4%)	0.9098
Home oxygen therapy	4 (2.7%)	2 (1.9%)	2 (4.9%)	0.3107
previous medication**	118 (79.2%)	87 (80.6%)	31 (75.6%)	0.5098
Symptoms				
Dyspnea	102 (68.5%)	77 (71.3%)	25 (61.0%)	0.2820
Tachypnea	83 (55.7%)	63 (58.3%)	20 (48.8%)	0.2977
Fatigue	63 (42.3%)	49 (45.4%)	14 (34.1%)	0.2182
Hipo-/Anosmia	6 (4.0%)	3 (2.8%)	3 (7.3%)	0.2108
Disgeusia	4 (2.7%)	3 (2.8%)	1 (2.4%)	0.9098
Sorethroat	16 (10.7%)	12 (11.1%)	4 (9.8%)	0.8130
Fever	63 (42.3%)	45 (41.7%)	18 (43.9%)	0.8067
Cough	63 (42.3%)	47 (43.5%)	16 (39.0%)	0.6961
Vomiting	11 (7.4%)	8 (7.4%)	3 (7.3%)	0.6227
Diarrhea	16 (10.7%)	14 (13.0%)	2 (4.9%)	0.9851
Arthromyalgy	8 (5.4%)	7 (6.5%)	1 (2.4%)	0.1566
Synkope	4 (2.7%)	4 (3.7%)	0 (0.0%)	0.3316
Chest pain	5 (3.4%)	3 (2.8%)	2 (4.9%)	0.2143
Headache	7 (4.7%)	5 (4.6%)	2 (4.9%)	0.5281
Rhinitis	6 (4.0%)	5 (4.6%)	1 (2.4%)	0.9494
O2SAT < 92%	74 (49.7%)	56 (51.9%)	18 (43.9%)	0.5467
Laboratory results at admission to ICU				
Leukocytes (/nl)	9.42 ± 4.78	9.08 ± 4.61	10.38 ± 5.17	0.1658
Lymphocytes (/nl)	0.88 ± 0.57	0.85 ± 0.59	0.94 ± 0.5	0.4813
Thrombocytes (/nl)	228.84 ± 114.48	229.07 ± 119.38	234.54 ± 95.71	0.8075
Hemoglobin (g/dl)	12.44 ± 2.38	12.53 ± 2.38	12.17 ± 2.4	0.4505
CRP (mg/dl)	17.94 ± 24.81	18.96 ± 28.36	15.1 ± 9.17	0.4321
Creatinine (mg/dl)	1.6 ± 1.84	1.45 ± 1.09	2.03 ± 3.07	0.1108
Bilirubin (mg/dl)	0.73 ± 0.8	0.75 ± 0.89	0.66 ± 0.43	0.5752
Therapy				
Highflow nasalcannula	109 (73.2%)	81 (75.0%)	28 (68.3%)	0.4127
Non-invasive mechanical ventilation	108 (72.5%)	79 (73.1%)	29 (70.7%)	0.7699
Invasive mechanical ventilation	148 (99.3%)	107 (99.1%)	41 (100.0%)	0.5396
Tracheotomy	48 (32.2%)	25 (23.1%)	23 (56.1%)	0.0001*
Vasoactive treatment	149 (100.0%)	108 (100.0%)	41 (100.0%)	1.0000
Cytokine adsorption therapy	36 (24.2%)	32 (29.6%)	4 (9.8%)	0.0112*
Adjuvant therapy on ECMO or mechanical ventilation	144 (96.6%)	104 (96.3%)	40 (97.6%)	0.7042
Neuromuscular blockage	66 (44.3%)	51 (47.2%)	15 (36.6%)	0.2460
Prone positioning	117 (78.5%)	81 (75.0%)	36 (87.8%)	0.0903
Nitrite oxide or prostacyclin	1 (0.7%)	0 (0.0%)	1 (2.4%)	0.1048
Renal replacement therapy	81 (54.4%)	62 (57.4%)	19 (46.3%)	0.2286
COVID19 treatment				
Use corticoids during admission	23 (15.4%)	18 (16.7%)	5 (12.2%)	0.5032
Use corticoids during ICU	135 (90.6%)	101 (93.5%)	34 (82.9%)	0.0482*
Immunsupressants	18 (12.1%)	15 (13.9%)	3 (7.3%)	0.2747
Antiviral drugs	60 (40.3%)	45 (41.7%)	15 (36.6%)	0.5752
Remedesivir	51 (34.2%)	40 (37.0%)	11 (26.8%)	0.2438
Tocilizumab	15 (10.1%)	14 (13.0%)	1 (2.4%)	0.0571
Hydrocychloroquin	9 (6.0%)	4 (3.7%)	5 (12.2%)	0.0525

Comparison between survivors and non-survivors.

Data are presentend as mean ± STD, unless otherwise indicated.

*SOFA* sepsis-related organ failure assessment score, *CRP* C-reactive protein, *ICU* intensive care unit, *ECMO* extracorporeal membrane oxygenation, *O2SAT* oxygen saturation at admission.

*p < 0.05, ** detailed medication list is shown in Supplement Table 1.

### Differences between ECMO and non-ECMO patients

**Figure 5:**
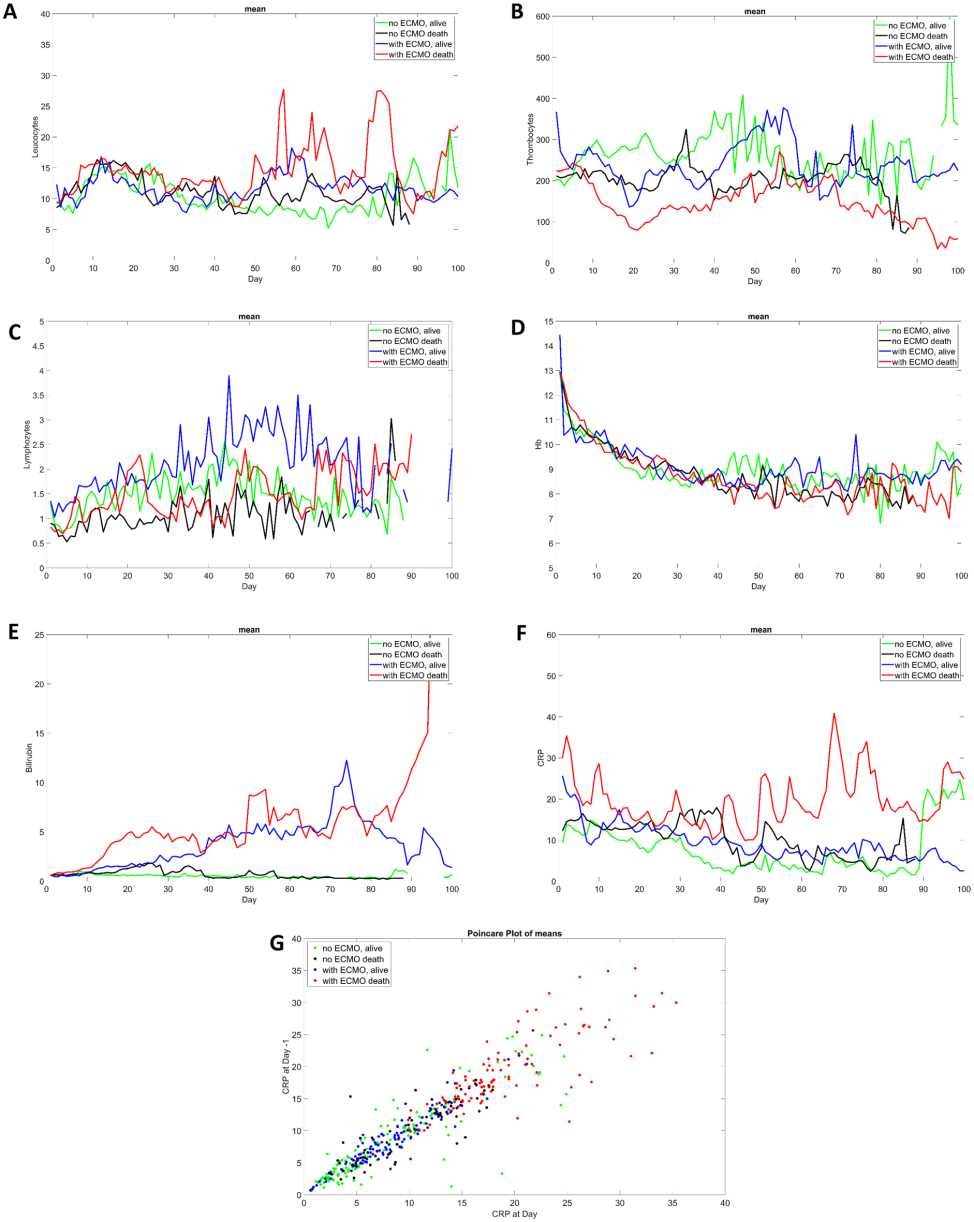
(**A**–**F**): some laboratory parameters over time (mean) (**G**): daily change of CRP at day t_i_-1 against day t_i_ as Poincaré plot.

Table 2 displays the parameters for non-ECMO (n = 99) and ECMO patients (n = 50). There were some significant differences between both groups. ECMO patients were on average 14 years younger and had less comorbidities compared to non-ECMO patients, especially cardiovascular diseases e.g. arterial hypertension, dyslipidemia and heart disease. As shown in Supplementary Table 2 previous medication, especially cardiovascular drugs, antidepressants and opioids were more often observed in the non-ECMO group. Regarding the symptoms, ECMO patients had significantly more dysgeusia, arthromyalgia and rhinitis. ECMO patients had higher leukocyte and CRP levels in the blood samples at admission, but also during hospital stay as shown in Fig. 5**.** For comparability reasons ventilatory parameters were shown one day after intubation. In ECMO patients we observed significantly higher levels of positive end expiratory pressure (PEEP), peak inspiratory pressure (PIP) and fraction of inspired oxygen (FiO_2_), but lower respiratory rates and tidal volumes compared to non-ECMO patients. Regarding treatment before intubation a higher percentage of patients in the ECMO group received high flow nasal cannula treatment. Significantly more cytokine adsorption and renal replacement therapy were carried out among the ECMO group. COVID-19 related medication treatments were comparable in both groups except for corticosteroid and Tocilizumab use with a higher frequency in the ECMO group.

**Table 2:** Basic characteristics of studied patients.

	ECMO 50/149 (33.6%)	Non-ECMO 99/149 (66.4%)	*p* value
Age (median), years (min–max)	58 (24–80)	72 (42–90)	< 0.0001*
Male	37 (74.0%)	58 (58.6%)	0.0653
Dead	40 (80.0%)	68 (68.7%)	0.1462
Comorbidities	47 (94.0%)	99 (100%)	0.0136*
Hypertension	29 (58.0%)	78 (78.8%)	0.0075*
Dyslipidemia	11 (22.0%)	38 (38.4%)	0.0448*
Diabetes mellitus	14 (28.0%)	40 (40.4%)	0.1388
Obesity	33 (66.0%)	62 (62.6%)	0.6882
Current smoker	3 (6.0%)	4 (4.0%)	0.5964
Renal insufficiency	4 (8.0%)	25 (25.3%)	0.0118*
Allergies	7 (14.0%)	16 (16.2%)	0.7323
Heart disease	9 (18.0%)	47 (47.5%)	0.0004*
Atrial fibrillation	1 (2.0%)	25 (25.3%)	0.0003*
Cerebrovascular disease	3 (6.0%)	11 (11.1%)	0.3159
Autoimmun disease	1 (2.0%)	0 (0.0%)	0.1601
Connective tissue disease	1 (2.0%)	0 (0.0%)	0.1601
Liver disease	1 (2.0%)	2 (2.0%)	0.9934
Any cancer	3 (6.0%)	12 (12.1%)	0.2439
Immunsupression condition	2 (4.0%)	2 (2.0%)	0.4835
Home oxygen therapy	0 (0.0%)	4 (4.0%)	0.1517
previous medication**	30 (60.0%)	88 (88.9%)	< 0.0001*
Symptoms			
Dyspnea	38 (76.0%)	64 (64.6%)	0.1612
Tachypnea	32 (64.0%)	51 (51.5%)	0.1494
Fatigue	22 (44.0%)	41 (41.4%)	0.7648
Hipo-/Anosmia	4 (8.0%)	2 (2.0%)	0.0805
Disgeusia	4 (8.0%)	0 (0.0%)	0.0041*
Sorethroat	7 (14.0%)	9 (9.1%)	0.3641
Fever	23 (46.0%)	40 (40.4%)	0.5171
Cough	21 (42.0%)	42 (42.4%)	0.9609
Vomiting	2 (4.0%)	9 (9.1%)	0.2648
Diarrhea	8 (16.0%)	8 (8.1%)	0.1423
Arthromyalgy	7 (14.0%)	1 (1.0%)	0.0008*
Synkope	0 (0.0%)	4 (4.0%)	0.1517
Chest pain	2 (4.0%)	3 (3.0%)	0.7582
Headache	3 (6.0%)	4 (4.0%)	0.5964
Pain	2 (4.0%)	2 (2.0%)	0.4835
Rhinitis	5 (10.0%)	1 (1.0%)	0.0082*
O2SAT < 92%	28 (56.0%)	46 (46.5%)	0.2748
Laboratory results at admission to ICU			
Leukocytes (/nl)	10.89 ± 4.42	8.78 ± 4.81	0.0194*
Lymphocytes (/nl)	0.9 ± 0.61	0.87 ± 0.56	0.8543
Thrombocytes (/nl)	244.18 ± 100.77	224.59 ± 118.3	0.3631
Hemoglobin (g/dl)	12.8 ± 2.22	12.28 ± 2.44	0.2523
CRP (mg/dl)	27.57 ± 42.21	13.81 ± 8.4	0.0030*
Creatinin (mg/dl)	1.35 ± 0.87	1.7 ± 2.11	0.3186
Bilirubin (mg/dl)	0.82 ± 0.79	0.69 ± 0.8	0.3883
pH	7.41 ± 0.1	7.38 ± 0.12	0.3429
pO2 (mmHg)	38.53 ± 13.54	41.82 ± 13.31	0.2183
pCO2 (mmHg)	74.8 ± 28.19	76.76 ± 28.19	0.7800
Lactate (mmol/l)	1.83 ± 1.43	2.1 ± 1.43	0.5198
Ventilatory settings one day after intubation			
PEEP (mmHg)	13 ± 2.81	11.71 ± 2.53	0.0070*
PIP (mmHg)	27.12 ± 5.62	25.13 ± 5.59	0.0732*
Tidal volume (ml)	418.33 ± 183.92	487.37 ± 150.83	0.0329*
FiO_2_(%)	68.63 ± 25.16	59.21 ± 18.45	0.0199*
Respiratory rate/minute	16 ± 7.14	18.2 ± 3.73	0.0244*
Therapy			
High flow nasal cannula	43 (86.0%)	66 (66.7%)	0.0117*
Non-invasive mechanical ventilation	41 (82.0%)	67 (67.7%)	0.0652
Invasive mechanical ventilation	49 (98.0%)	99 (100.0%)	0.1601
Tracheotomy	21 (42.0%)	27 (27.3%)	0.0701
Vasoactive treatment	50 (100.0%)	99 (100.0%)	
Cytokine adsorption therapy	23 (46.0%)	13 (13.1%)	< 0.0001*
Adjuvant therapy on ECMO or mechanical ventilation	47 (94.0%)	97 (98.0%)	0.2053
Neuromuscular blockage	21 (42.0%)	45 (45.5%)	0.6909
Prone positioning	38 (76.0%)	79 (79.8%)	0.5969
Nitrite oxide or prostacyclin	1 (2.0%)	0 (0.0%)	0.1601
Renal Replacement therapy	38 (76.0%)	43 (43.4%)	0.0001*
COVID19 treatment			
Use corticoids during admission	8 (16.0%)	15 (15.2%)	0.8932
Use corticoids during ICU	50 (100.0%)	85 (85.9%)	0.0050*
Immunsupressants	9 (18.0%)	9 (9.1%)	0.1167
Antiviral drugs	20 (40.0%)	40 (40.4%)	0.9624
Remedesivir	18 (36.0%)	33 (33.3%)	0.7480
Tocilizumab	9 (18.0%)	6 (6.1%)	0.0221*
Hydrocychloroquin	1 (2.0%)	8 (8.1%)	0.1432

Comparison between ECMO patients and Non-ECMO patients.

Data are presentend as mean ± STD, unless otherwise indicated.

*ECMO* extracorporeal membrane oxygenation, *O2SAT* oxygen saturation at admission, *ICU* intensive care unit, *CRP* C-reactive protein, *PEEP* positive end expiratory pressure, *PIP* peak inspiratory pressure, *FiO*_*2*_ fraction of inspired oxygen.

*p < 0,05, **detailed medication list is shown in Supplement Table 2. Significant values are in [bold].

### Resource requirements and complications

In Table 3 resource requirements are shown for ECMO and non-ECMO patients. A significant transfusion of blood-derived components was observed. Most apparently, transfusion of thrombocytes was necessary 12-fold more often among the ECMO patients, while overall bleeding complications occurred more than 4 times more often among the ECMO group. Onset of atrial fibrillation, but not atrial flutter during ICU stay was significantly increased in non-ECMO patients.

**Table 3:** Complications and resource use during hospital stay: ECMO patients compared to Non-ECMO patients.

Overall Complications	ECMO 50/149 (33.6%)	Non-ECMO 99/149 (66.4%)	*p* value
Cardiovascular complications			
Heart failure at admission	1 (2.0%)	4 (4.0%)	0.5170
Endocarditis	0 (0.0%)	1 (1.0%)	0.4792
Arrythmia	13 (26.0%)	41 (41.4%)	0.0653
* SVES*	0 (0.0%)	1 (1.0%)	0.4792
* VES*	1 (2.0%)	0 (0.0%)	0.1601
* Atrial fibrillation*	5 (10.0%)	30 (30.3%)	0.0056*
* Atrail flutter*	2 (4.0%)	0 (0.0%)	0.0455*
* SVT*	3 (6.0%)	4 (4.0%)	0.5964
* VT*	0 (0.0%)	3 (3.0%)	0.2164
* Ventricular fibrillation*	0 (0.0%)	0 (0.0%)	1.0000
* Ventricular flutter*	0 (0.0%)	0 (0.0%)	1.0000
Cardiopulmonary resuscitation	9^#^ (18.0%)	21 (21.2%)	0.7229
Embolic event	10 (20.0%)	14 (14.1%)	0.3617
Bleeding complications			
Any relevant bleeding	34 (68.0%)	15 (15.2%)	< 0.0001*
Hemoptysis	2 (4.0%)	0 (0.0%)	0.0455*
Anemia	48 (96.0%)	88 (88.9%)	0.1484
Thrombocytopenia	35 (70.0%)	43 (43.4%)	0.0020*
Other organ complications			
Renal failure	33 (66.0%)	52 (52.5%)	0.1182
Liver failure	9 (18.0%)	8 (8.1%)	0.0730
Pneumonia	44 (88.0%)	89 (89.9%)	0.7259
Sepsis	43 (86.0%)	72 (72.7%)	0.0691
SIRS	43 (86.0%)	68 (68.7%)	0.0220*
Pleural effusion	14 (28.0%)	33 (33.3%)	0.5115
Pneumothorax	10 (20.0%)	11 (11.1%)	0.1428
Reintubation	2 (4.0%)	9 (9.1%)	0.2648
Resource use			
Transfusion	47 (94%)	62 (62.6%)	< 0.0001*
Transfusion of one or more EC	47 (94%)	54 (54.5%)	< 0.0001*
Transfusion of one or more TC	19 (38%)	3 (3.0%)	< 0.0001*
Transfusion of one or more FFP	11 (22%)	19 (19.2%)	0.6889
Erythrocyte concentrates	12.16 ± 12.08	2.72 ± 4.48	< 0.0001*
Thrombocyte concentrates	1.50 ± 3.02	0.12 ± 0.93	< 0.0001*
Plasma concentrate	3.48 ± 10.12	0.69 ± 1.69	0.0084*

Data are presentend as mean ± STD, unless otherwise indicated.

*SVES* supraventricular extrasystole, *VES* vetricular extrasystole, *SVT* supravetricular tachycardia, *VT* ventricular tachycardia, *SIRS* systemic inflammatory response syndrome, *EC* erythrocyte concentrate, *TC* thrombocte concentrate, *FFP* fresh frozen plasma.

*p < 0.05.

^#^CPR occured during ECMO period. When VV-ECMO therapy failed a switch to VA-ECMO was not performed. Significant values are in [bold].

### Differences between ECMO survivors and non-survivors

Among the ECMO patients the mortality rate was high as 80% with no significant age differences. Table 4 displays the characteristics for ECMO survivors (n = 10) and non-survivors (n = 40). For instance, a portion of 80% (n = 8) of ECMO survivors were tracheotomized, compared to only one third (n = 13) of deceased ECMO patients.

**Table 4:** Basic characteristics of studied patients.

Category	ECMO non-survivors 40/50 (80%)	ECMO survivors 10/50 (20%)	*p* value
Age (median), years (min–max)	59 (36–88)	53 (24–74)	0.1307
Male	30 (75.0%)	7 (70%)	0.7532
SOFA score	11.88 (7–20)	10.78 (7–13)	0.2013
Comorbidities	38 (95.0%)	9 (90%)	0.5609
Hypertension	23 (57.5%)	6 (60%)	0.8889
Dyslipidemia	8 (20.0%)	3 (30%)	0.5047
DM	12 (30.0%)	2 (20%)	0.5384
Obesity	27 (67.5%)	6 (60%)	0.6622
Current smoker	1 (2.5%)	2 (20%)	0.0377*
Renal insufficiency	4 (10.0%)	0 (00%)	0.3069
Allergies	6 (15.0%)	1 (10%)	0.6909
Heart disease	7 (17.5%)	2 (20%)	0.8576
Atrial fibrillation	1 (2.5%)	0 (00%)	0.6221
Cerebrovascular disease	3 (7.5%)	0 (00%)	0.3820
Autoimmun disease	1 (2.5%)	0 (00%)	0.6221
Connective tissue disease	1 (2.5%)	0 (00%)	0.6221
Liver disease	1 (2.5%)	0 (00%)	0.6221
Any cancer	3 (7.5%)	0 (00%)	0.3820
Immunsupression condition	2 (5.0%)	0 (00%)	0.4806
Home oxygen therapy	0 (0.0%)	0 (00%)	
Symptoms			
Dyspnea	31 (77.5%)	7 (70%)	0.6279
Tachypnea	25 (62.5%)	7 (70%)	0.6663
Fatigue	18 (45.0%)	4 (40%)	0.7812
Hipo-/Anosmia	3 (7.5%)	1 (10%)	0.7994
Disgeusia	3 (7.5%)	1 (10%)	0.7994
Sorethroat	6 (15.0%)	1 (10%)	0.6909
Fever	19 (47.5%)	4 (40%)	0.6780
Cough	18 (45.0%)	3 (30%)	0.4004
Vomiting	2 (5.0%)	0 (00%)	0.4806
Diarrhea	8 (20.0%)	0 (00%)	0.1279
Athromyalgia	6 (15.0%)	1 (10%)	0.6909
Syncopy	0 (0.0%)	0 (00%)	1.0000
Chest pain	1 (2.5%)	1 (10%)	0.2885
Headache	3 (7.5%)	0 (00%)	0.3820
Rhinitis	5 (12.5%)	0 (00%)	0.2473
O2SAT < 92%	22 (55.0%)	6 (60%)	0.7812
Laboratory results at admission to ICU			
Leucocytes	10.38 ± 4.38	12.94 ± 4.38	0.1454
Lymphocytes	0.82 ± 0.65	1.13 ± 0.45	0.2446
Thrombocytes	233.03 ± 97.52	288.75 ± 107.8	0.1647
Hemoglobin	12.71 ± 2.31	13.14 ± 1.93	0.6344
CRP	30.66 ± 46.4	15.19 ± 13.15	0.3605
Creatinin	1.48 ± 0.92	0.85 ± 0.31	0.0666
Bilirubin	0.83 ± 0.87	0.76 ± 0.41	0.8078
Therapy			
Highflow_nasalcannula	35 (87.5%)	8 (80%)	0.5505
Non-invasive mechanical ventilation	34 (85.0%)	7 (70%)	0.2788
Invasive mechanical ventilation	39 (97.5%)	10 (100%)	0.6221
Tracheotomy	13 (32.5%)	8 (80%)	0.0058*
Intubation to tracheotomy (days)	7.45 ± 12.02	19.4 ± 13.28	0.0083*
Vasoactive treatment	40 (100.0%)	10 (100%)	1.0000
Cytokine adsorption therapy	21 (52.5%)	2 (20%)	0.0673
Adjuvant therapy on ECMO	38 (95.0%)	9 (90%)	0.5609
Neuromuscular blockage	16 (40.0%)	5 (50%)	0.5758
Prone positioning	29 (72.5%)	9 (90%)	0.2554
Nitrite oxide or prostacyclin	0 (0.0%)	1 (10%)	0.0443*
Renal Replacement therapy	32 (80.0%)	6 (60%)	0.1927
COVID19 treatment			
Use corticoids during admission	6 (15.0%)	2 (20%)	0.7067
Use corticoids during ICU	40 (100.0%)	10 (100%)	1.0000
Immunsupressants	9 (22.5%)	0 (00%)	0.1015
Antiviral drugs	16 (40.0%)	4 (40%)	1.0000
Remedesivir	15 (37.5%)	3 (30%)	0.6663
Tocilizumab	9 (22.5%)	0 (00%)	0.1015
Hydrocychloroquin	0 (0.0%)	1 (10%)	0.0443*

Comparison between ECMO survivors and ECMO non-survivors.

Data are presentend as mean ± STD, unless otherwise indicated.

*ECMO* extracorproreal membrane oxygenation, *SOFA* sepsis-related organ failure assessment score, *ASS* acetylsalicic acid, *ACEI* angiotensin-converting enzyme inhibitor, *ARB* angiotensin-II-receptor blocker, *Ca-Antagonist* calcium-antagonist, *PPI* proton-pump inhibitor, *NSAR* non-steroidal antiinflammatory drugs, *O2SAT* oxygen saturation at admission, *CRP* c-reactive protein, *ICU* intensive care unit.

*p < 0.05. Significant values are in [bold].

### Laboratory courses dependent on outcome and ECMO support

In Fig. 5 the daily obtained laboratory results of C-reactive protein (CRP), leukocytes, lymphocytes, erythrocytes, thrombocytes and bilirubin were summarized. Noticeable differences are the undulating CRP in deceased ECMO patients. This observation is also shown as a Poincare plot. The diffusivity of distribution underlines the frequent changes of CRP during ICU stay.

### Comparison of high- and low-volume ECMO centers

Dependent on annual ECMO treatment numbers we categorized high- and low volume ECMO centers (cut-off: 20 ECMO treatments) as shown in Table 5. Of the four hospitals, three ICUs perform less than 20 ECMO treatments per year. More than 50% of patients treated in the high-volume center got ECMO support, whereas in the low-volume center only 25% of patients were treated with ECMO. Mortality rates did not differ significantly, but there was a tendency of higher mortality in the high-volume center compared to the low-volume centers. Significantly higher percentages of male sex and both SOFA (Sepsis-related organ failure assessment) and RESP (Respiratory Extracorporeal Membrane Oxygenation Survival Prediction) score were observed in the high-volume center. There was no significant difference between ECMO centers regarding the PRESET score (PREdiction of Survival on ECMO Therapy Score)^21,22^. Cytokine adsorption therapy was performed significantly more in the high-volume center. The amount of stroke as the only significant complication during ECMO support was higher in the high-volume center. Regarding laboratory results, lower values for lymphocytes, base excess, sodium, calcium, and chloride were observed in the high-volume center, but pleural effusion was detected significantly more in the low-volume centers.

**Table 5:** Comparison of clinical characteristics of ECMO centres with high- and low volume ECMO use.

	ICU > 20 ECMO patients/year (1 ICU)	ICU < 20 ECMO patients/year (3 ICUs)	*p* value
Number of all patients	39	110	
Number of ECMO patients (%)	22 (56.41%)	28 (25.46%)	0.0004*
Age all patients, years (median)	64	68	0.1002
Age ECMO patients, years (median)	59	58	0.7484
Male sex all patients (%)	84.62%	56.36%	0.0015*
Male sex ECMO patients (%)	90.90%	60.71%	0.0152*
Mortality—all patients	28 (71.79%)	80 (72.73%)	0.9115
Mortality—ECMO patients	20 (90.91%)	20 (71.43%)	0.0907
ECMO treatment before COVID pandemic	Yes	Yes	
Center or staff experience (years)	30 y	6–25 y	
Cytokine adsorption therapy	41%	18.18%	0.0040*
Days on ECMO support	9.28 ± 11.64	6.12 ± 14.44	0.2183
SOFA Score at ECMO initiation	12.44	10.64	0.0073*
RESP-Score^1^ at ECMO initiation	-1.1	0.4	0.0146*
PRESET Score^2^ at ECMO initiation	8.09	7.81	0.6845
Complications during ECMO support	73%	57%	0.2635
ECMO circuit change	36%	25%	0.3944
Intravasal haemolysis	9%	14%	0.5838
Clogged circuit requiring change	5%	7%	0.7081
Repeat ECMO after decannulation	0%	0%	–
Severe thrombocytopenia	0%	4%	0.3809
Heparin-induced thrombocytopenia	0%	14%	0.0667
Massive haemorrhage	55%	46%	0.5780
Stroke	27%	4%	0.0217*
Cannula infection	5%	0%	0.2635
Pulmonary embolism	9%	11%	0.8531
Cardiac arrest	14%	18%	0.6934
Tracheostomy	55%	46%	0.5780
Pneumothorax	27%	14%	0.2635
Ventilator-associated pneumonia	91%	79%	0.2462
Bacteremia	73%	79%	0.6393
Other organ complications of ECMO patients			
Renal failure	68.18%	64.29%	0.7783
Liver failure	9.09%	25.00%	0.1521
Pneumonia	90.91%	85.71%	0.5838
Sepsis	77.27%	92.86%	0.1196
SIRS	77.27%	92.86%	0.1196
Reintubation#	4.55%	3.57%	0.8649
Pleural effusion	13.64%	39.29%	0.0460*
Laboratory results at ECMO initiation			
Leucocytes (/nl)	19.9 ± 11.85	17.77 ± 6.6	0.5119
Lymphocytes (/nl)	0.84 ± 0.8	1.6 ± 0.47	0.0347*
Thrombocytes (/nl)	207.64 ± 95.38	212.46 ± 43	0.8717
Hemoglobin (g/dl)	11.37 ± 1.7	9.99 ± 6.4	0.0178*
D Dimer	2.47 ± 1.27	9.95 ± 2.65	0.2282
CRP	27.15 ± 28.48	19.41 ± 0.18	0.2301
Creatinine	1.7 ± 1.5	1.14 ± 0.33	0.0906
Procalcitonine	8.14 ± 13.02	2.89 ± 0.08	0.1215
Bilirubine	1.27 ± 0.69	0.98 ± 0.3	0.2225
pH	7.27 ± 0.18	7.35 ± 7.14	0.0599
pCO2 in mmHg	50.27 ± 9.55	53.04 ± 33.8	0.4234
pO2 in mmHg	72.73 ± 26.09	77.28 ± 54.2	0.4849
sO2	92.23 ± 5.02	92.61 ± 79.6	0.7874
HCO3 mmol/l	22.56 ± 9.56	26.73 ± 12.2	0.0608
Base excess (mmol/l)	(−) 2.95 ± 12.32	2.79 ± (-15.6)	0.0417*
Potassium (mmol/l)	5.25 ± 1.04	4.76 ± 3.5	0.0813
Sodium (mmol/l)	139.91 ± 3.96	144.27 ± 134	0.0014*
Calcium (mmol/l)	1.03 ± 0.15	1.18 ± 0.91	0.0003*
Chloride (mmol/l)	106.95 ± 2.82	109.86 ± 104	0.0299*
Glucose (mg/dl)	188.05 ± 85.55	202.08 ± 117	0.5093
Lactate (mmol/l)	3.15 ± 4.43	3.28 ± 1	0.9179
Resource use of ECMO patients			
Transfusion	90.91%	96.43%	0.4250
Transfusion with EC	90.91%	96.43%	0.4250
Transfusion with TC	45.45%	32.14%	0.3459
Transfusion with FFP	4.55%	35.71%	0.0075*
EC per patient	9.64 ± 6.38	14.14 ± 14.96	0.1932
TC per patient	1.86 ± 2.55	1.21 ± 3.36	0.4559
FFP per patient	0.05 ± 0.21	6.18 ± 12.99	0.0320*

Data are presentend as mean ± STD, unless otherwise indicated.

*SOFA* sepsis-related organ failure assessment, *RESP* respiratory extracorporeal membrane oxygenation survival prediction, *PRESET* PREdiction of survival on ECMO therapy, *SIRS* systemic inflammatory response syndrome, *EC* erythrocyte concentrate, *TC* thrombocte concentrate, *FFP* fresh frozen plasma.

^1^score ranges from below − 6 (lower limt value) and above + 6 (upper limit value). A higher value indicates a better prognosis.

^2^score ranges from 0 to 15. A higher value indicates a higher mortality.

*p < 0.05.

^#^All reintubation cases occured after ECMO removal.

## Discussion

This is the first analysis of patients with very severe COVID-19 infection during the first three waves in the Middle Ruhr Region, Germany’s largest and Europe’s fourth largest urban area. In this retrospective observational cohort study, we found that patients admitted to the ICU and treated with IMV due to severe COVID-19 infection—independent of ECMO use—had a high mortality rate. This observation is in accordance with prior published German registry data and confirms the higher mortality of German patients with and without ECMO support in comparison to many other European countries^15,16,23^. As reported in a large cohort study, advanced age is a strong factor associated with COVID-19 related death^24^. Hence, a possible explanation for the higher mortality observed in the present study could be the age structure of the study population. The mean age of 67 years is comparable to other reported studies^25,26^, but the wide distribution of the patients with a percentage of 49% which are at least 70 years old or older resulted in a mortality rate of 89%. Patients 80 years or older contributed 15% to the study population and presented a particularly high mortality rate of 95%. In conclusion, the reported high mortality of 72.5% in our study population is predominated by the very high mortality of the elderly admitted to ICU for IMV. Furthermore, due to the ubiquitary availability of ECMO support in Germany the use of this tool, till now, is not limited by older age. In our population the mean age of ECMO patients was 58 years, which is significantly higher than that in other European countries ranging from 49 to 52 years^7,11,14^. Similarly, the age structure among the ECMO patients was remarkably older. Nearly half of the ECMO patients aged at least 60 years or older with a mortality rate of 83%. Considering only patients 50 years old or younger (52% portion) the mortality rate is very high with 80% and still not comparable to mortality rates of other European countries. On the one hand the high mortality rate is explainable by elderly patients treated on ICU, on the other hand also young patients especially with ECMO support had high mortality rates. This observation of a remarkable high mortality rate despite young age of ECMO group has to be observed concerning different mortality rates of German patients to other countries. A possible explanation could be an extension of inclusion criteria of ECMO treatment for potential desperate clinical cases due to the sparsely regulated and quite unlimited possibility of ECMO availability in Germany.

One contributing factor explaining the higher mortality for ICU patients could be the reported prolongation of IMV interval before ECMO initiation. Supady et al. reported a slightly, but significantly better survival of patients with a shorter interval from IMV to ECMO initiation with a cut-off at 7 days (46.8% vs. 43.0%)^27^. Furthermore, in a meta-analysis the pre-hospitalization and intubation periods were longer in intubated non-survivors and ECMO patients than in intubated survivors^28^. In our study we observed that ECMO non-survivors have a shorter interval of IMV to ECMO initiation in comparison to ECMO survivors. But regarding the pre-hospitalization periods in ECMO deaths we observed on average a 2 days longer interval of symptom onset to hospital admission in comparison to ECMO survivors. Also in the non-ECMO patients, the deaths had a longer pre-hospitalization period on average 2.3 days in comparison to non-ECMO survivors. Obviously in our study population the pre-hospitalization period seems to play an important role in higher mortality rates independent of ECMO support.

Additionally, a detailed comparison of high- and low-volume ECMO centers in this study did not show a volume-outcome relationship. In the international ELSO (Extracorporeal Life Support Organization) registry higher annual hospital ECMO volume was associated with lower mortality and consequently the recommendation of performing at minimum 20 ECMO procedures per year^29,30^. On the contrary, German registry data with 29 929 ECMO patients could not confirm a clear linear volume-outcome relationship. Indeed, a higher mortality rate was observed in centers performing less than 6 procedures per year, but the authors highlight on ‘’irregular’’ mortality outcomes with a higher mortality in high-volume centers^31^. The authors explained this increased mortality by a larger volume of complex patients and a higher proportion of patients who were already referred from other hospitals. This is in accordance with our observation with a high mortality rate in the high-volume center with more severely ill patients admitted for ECMO support, indicated by higher SOFA and RESP scores. Interestingly the percentage of male sex was 90% in the high-volume center compared to only 60% in the low-volume centers. Also this fact explains higher mortality rates, due to the reported excess mortality for male patients during the COVID-19 pandemic^32–34^. The utilization of cytokine adsorption treatment in the high-volume center could also contribute to a higher mortality as discussed below.

Supported by the observation of extensively increased cytokine concentrations, the attenuation of the uncontrolled cytokine response is one of the evolving treatment strategies of severe COVID-19^10,35,36^. In 2020, the US Food and Drug Administration authorized an emergency use for the CytoSorb adsorber for treatment of COVID-19. Unexpectedly, a later randomized and controlled trial with 34 patients observed a negative effect of cytokine adsorption treatment on the survival especially in ECMO patients^37^. With all the limitations of a retrospective study, our data supports this finding since cytokine adsorption therapy was used significantly more often in non-survivors compared to survivors in general and, at least numerically (*p* = 0.0673) among the ECMO patients. Due to the sample size of the above mentioned prospective study, further studies with larger data bases should be performed to clarify the effect of cytokine adsorption.

Interestingly, the prescription of antidepressants (pre-hospital medication) was significantly higher in survivors. In a multicenter cohort study analyzing electronic health records of 83 584 patients diagnosed with COVID-19, including 3401 patients who were prescribed Selective Serotonin Reuptake Inhibitors (SSRI), a reduced relative risk of mortality was found to be associated with the use of SSRIs compared to patients who were not prescribed SSRIs^38^. In another multicenter observational retrospective study with 7230 patients, antidepressant use (SSRI and non-SSRI) was significantly associated with lower risk of intubation or death among adult patients hospitalized for COVID-19^39^. The hypothesis of these beneficial effects is a regulating influence of antidepressants on several proinflammatory cytokines suggested to be involved in the development of severe COVID-19, and even direct antiviral effects^40^. A recent placebo-controlled randomized trial found that patients assigned to fluvoxamine, a SSRI, showed a lower risk of hospitalization, without effects on the mortality^41^.

Based on the availability of daily laboratory data for every ICU patient in this study, we categorized the patients into four groups depending on ECMO and survival and were able analyze multiple serum markers. An important observation regarding the course of daily CRP, a marker of acute inflammation, is an undulation of serum levels in deceased ECMO patients, which was outlined in this paper by a scattering in the Poincare plot. Previous studies showed that higher levels of CRP are associated with higher mortality and linked to disease progression and severity^42–44^. Whether higher or undulating CRP levels are caused by a maintaining infectious status or recurrent complications such as nosocomial infections or other severe complications has to be elucidated in further analyses. Another interesting biomarker is bilirubin, a marker of liver function integrity, which has been shown to correlate to severity and mortality in COVID‐19 patients^45,46^. In our cohort the deceased patients with ECMO support presented a final massive increase in bilirubin levels, probably reflecting the multiple organ failure consistent with data derived from septic patients^47^. A lower lymphocyte count has been associated with an increased disease severity and mortality in COVID-19^8,48^. During ECMO support decreases in the number of lymphocytes is common and therefore it was hypothesized that repletion of lymphocytes could be a way of recovery in COVID-19^48,49^. Our data support this hypothesis by showing a trend of higher lymphocyte counts during ICU treatment in surviving patients with an obvious upstroke in lymphocyte counts especially in ECMO survivors. Alternatively, other possible reasons of bilirubin rise should be taken into account as consequences of bleeding, hemolysis related to ECMO circuit or secondary effects of massive transfusions.

Finally the higher bleeding risk and excess mortality raises particular concern during ECMO support. Compared to non-ECMO patients the utilization of 12-fold more thrombocyte concentrates in ECMO patients in our study population is remarkable. There are only a few studies reporting distinct amounts of thrombocyte concentrate transfusion, one study reported a fivefold higher utilization and another observed the use of thrombocyte concentrate in 13% of ECMO patients^14,50^. There are several factors that explain these differences. On the one hand, the present study included a larger and, as discussed above, an older population. On the other hand, Schmidt and colleagues referred only to the first wave of the COVID-19 pandemic, whereas our data cohort covers two further pandemic waves. And doubtless, the mortality and severity of COVID-19 worsened during the pandemic^11^ which probably resulted in higher utilization of blood-derived products.

Our study has several limitations. In this retrospective study design we performed a day-to-day follow-up, but the number of patients was too low to perform valid predictive statistical models, even less for the ECMO patients. Therefore we outlined patients’ characteristics only by descriptive statistics. We included all the critically ill patients from four different hospitals with consequently different standards of care and different diagnostic and therapeutic tools.

## Conclusion

Unvaccinated people or breakthrough infections are ongoing challenges for the health care systems in the future since the COVID-19 pandemic continues to be severe, particularly in certain population groups. Despite a desired beneficial impact of ECMO support among patients with COVID-19 the mortality rate is very high despite large resource employment. Therefore, it is crucial to reveal factors that predict the outcome of ECMO support in patients with severe COVID-19, to standardize the setting of ECMO support, and to define parameters that clearly indicate and contraindicate its initiation. According to our data, advanced age and longer pre-hospitalization periods as well as certain laboratory parameters and the use of cytokine absorption therapy may explain worse outcomes. Prospective and controlled, favorably multi-center trials are mandatory to further elucidate the role of ECMO therapy.

## Supplementary Information

Supplementary Information 1.

Supplementary Information 2.

Supplementary Information 3.

Supplementary Information 4.

## Data Availability

The datasets used for the analysis in the current study are available from the corresponding author on reasonable request. Assem Aweimer and Lea Petschulat had full access to all the data in the study and take responsibility for the integrity of the data and the accuracy of the data analysis.
