# Publisher Correction: Mortality rates of severe COVID-19-related respiratory failure with and without extracorporeal membrane oxygenation in the Middle Ruhr Region of Germany

**DOI:** 10.1038/s41598-023-33475-7

**Published:** 2023-04-20

**Authors:** Assem Aweimer, Lea Petschulat, Birger Jettkant, Roland Köditz, Johannes Finkeldei, Johannes W. Dietrich, Thomas Breuer, Christian Draese, Ulrich H. Frey, Tim Rahmel, Michael Adamzik, Dirk Buchwald, Dritan Useini, Thorsten Brechmann, Ingolf Hosbach, Jürgen Bünger, Aydan Ewers, Ibrahim El‑Battrawy, Andreas Mügge

**Affiliations:** 1 Department of Cardiology and Angiology, BG University Hospital Bergmannsheil, Ruhr-Universität Bochum, Bürkle‑de‑La‑Camp‑Platz 1, 44789 Bochum, Germany; 2 Institute for Prevention and Occupational Medicine of the German Social Accident Insurance, Institute of the Ruhr-Universität Bochum (IPA), Bochum, Germany; 3 Department of Endocrinology and Diabetes, BG University Hospital Bergmannsheil, Ruhr University of Bochum, Bochum, Germany; 4 Diabetes, Endocrinology and Metabolism Section, Medical Hospital I, Katholisches Klinikum Bochum, St Josef Hospital Bochum, Ruhr University Bochum, Bochum, Germany; 5 Department of Internal Medicine, Katholisches Klinikum Bochum, St. Josef-Hospital, Ruhr-University Bochum, Bochum, Germany; 6 Klinik für Anästhesiologie, Operative IntensivmedizinSchmerz‑und PalliativmedizinMarien Hospital Herne, Universitätsklinikum der Ruhr-Universität Bochum, Bochum, Germany; 7 Klinik für Anästhesiologie, Intensivmedizin und Schmerztherapie, Universitätsklinikum Knappschaftskrankenhaus Bochum, Bochum, Germany; 8 Department of Cardiothoracic Surgery, BG University Hospital Bergmannsheil, Ruhr-University Bochum, Bochum, Germany; 9 Gastroenterology and Hepatology, BG University Hospital Bergmannsheil, Bochum, Germany

Correction to: *Scientific Reports* 10.1038/s41598-023-31944-7, published online 29 March 2023

In Figure 5, panels E, F and G were omitted in the original version of this Article. The original Figure 5 and accompanying legend appear below.

**Figure 5:**
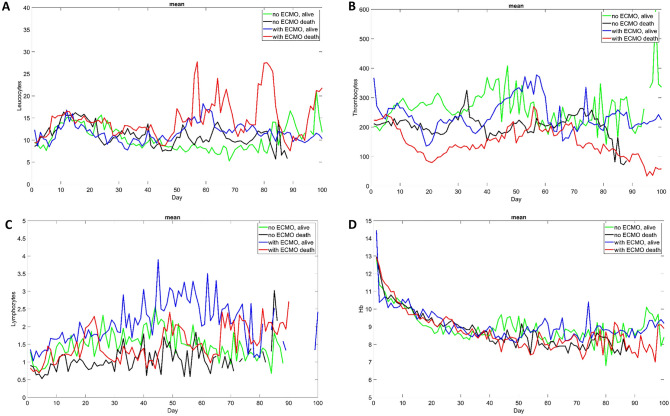
(**A**–**F**): some laboratory parameters over time (mean) (**G**): daily change of CRP at day t_i_ - 1 against day ti as Poincaré plot.

The original Article has been corrected.

