# Supplementary material for: Mortality rates of severe COVID-19-related respiratory failure with and without extracorporeal membrane oxygenation in the Middle Ruhr Region of Germany
Source: Sci Rep. 2023 Mar 29;13:5143. doi: 10.1038/s41598-023-31944-7 (PMC10054204; doi:10.1038/s41598-023-31944-7)
Supplement: Supplementary file 1 — Supplementary Information 1. [file 41598_2023_31944_MOESM1_ESM.docx]

***Supplementary Table 1****: Pre-medication of patient population, comparison survivors and non survivors*

|  | **All patients 149/149 (100%)** | **Non survivors 108/149 (72,5%)** | **survivors 41/149 (27,5%)** | **p Value** |
| --- | --- | --- | --- | --- |
| **Previous aspirin** | 44 (29,5%) | 34 (31,5%) | 10 (24,4%) | 0,4002 |
| **Antiplatelet** | 7 (4,7%) | 6 (5,6%) | 1/1 (2,4%) | 0,4254 |
| **Oral anticoagulant** | 26 (17,4%) | 21 (19,4%) | 5 (12,2%) | 0,3010 |
| **ACEI** | 51 (34,2%) | 40 (37,0%) | 11 (26,8%) | 0,2438 |
| **ARB** | 22 (14,8%) | 13 (12,0%) | 9 (22,0%) | 0,1294 |
| **Beta blockers** | 57 (38,3%) | 40 (37,0%) | 17 (41,5%) | 0,6224 |
| **Betaagonist inhaled** | 20 (13,4%) | 16 (14,8%) | 4 (9,8%) | 0,4220 |
| **Glucocorticoids inhaled** | 8 (5,4%) | 8 (7,4%) | 0 (0,0%) | 0,0741 |
| **Vitamin D suplement** | 12 (8,1%) | 7 (6,5%) | 5 (12,2%) | 0,2553 |
| **Benzodiacepines** | 2 (1,3%) | 2 (1,9%) | 0 (0,0%) | 0,3838 |
| **Antidepressant** | 16 (10,7%) | 7 (6,5%) | 9 (22,0%) | **0,0062*** |
| **Ca-Antagonists** | 42 (28,2%) | 31 (28,7%) | 11 (26,8%) | 0,8218 |
| **Diuretics** | 52 (34,9%) | 40 (37,0%) | 12 (29,3%) | 0,3777 |
| **Statins** | 43 (28,9%) | 32 (29,6%) | 11 (26,8%) | 0,7383 |
| **Thyroxin substitution** | 23 (15,4%) | 18 (16,7%) | 5 (12,2%) | 0,5032 |
| **Antikonvulsives** | 13 (8,7%) | 9 (8,3%) | 4 (9,8%) | 0,7852 |
| **Antidiabetics** | 31 (20,8%) | 22 (20,4%) | 9 (22,0%) | 0,8332 |
| **Insulin** | 24 (16,1%) | 20 (18,5%) | 4 (9,8%) | 0,1963 |
| **Antibiotics** | 8 (5,4%) | 4 (3,7%) | 4 (9,8%) | 0,1452 |
| **Uricostatics** | 15 (10,1%) | 12 (11,1%) | 3 (7,3%) | 0,4952 |
| **PPI** | 53 (35,6%) | 36 (33,3%) | 17 (41,5%) | 0,3579 |
| **Alpha antagonists** | 18 (12,1%) | 18 (16,7%) | 0 (0,0%) | **0,0051*** |
| **NSARs (**except ASS) | 19 (12,8%) | 13 (12,0%) | 6 (14,6%) | 0,6737 |
| **Opioids** | 17 (11,4%) | 7 (6,5%) | 10 (24,4%) | **0,0020*** |
| **Dopamin medication** | 6 (4%) | 6 (5,6%) | 0 (0,0%) | 0,1251 |
| **Vitamin supplements** | 10 (12,8%) | 13 (12,0%) | 6 (14,6%) | 0,6737 |
| **Antiarrhythmics** | 4 (2,7%) | 3 (2,8%) | 1 (2,4%) | 0,9098 |
| **Antihistaminics** | 16 (10,7%) | 12 (11,1%) | 4 (9,8%) | 0,8130 |

*ASS, acetylsalicylic acid; ACEI, Angiotensin-converting enzyme-inhibitors; ARB, angiotensin-II-blockers; NSAR, non-steroidal anti-inflammatory drugs*
