# Supplementary material for: Mortality rates of severe COVID-19-related respiratory failure with and without extracorporeal membrane oxygenation in the Middle Ruhr Region of Germany
Source: Sci Rep. 2023 Mar 29;13:5143. doi: 10.1038/s41598-023-31944-7 (PMC10054204; doi:10.1038/s41598-023-31944-7)
Supplement: Supplementary file 2 — Supplementary Information 2. [file 41598_2023_31944_MOESM2_ESM.docx]

***Supplementary Table 2:*** *Pre-medication of patient population, comparison of ECMO patients and non-ECMO (ventilated) patients*

|  | **ECMO 50/149 (33,6%)** | **Non-ECMO 99/149 (66,4%)** | **p value** |
| --- | --- | --- | --- |
| **Previous aspirin** | 9 (18,0%) | 35 (35,4%) | **0,0284*** |
| **Antiplatelet** | 2 (4,0%) | 5 (5,1%) | 0,7766 |
| **Oral anticoagulant** | 6 (12,0%) | 20 (20,2%) | 0,2156 |
| **ACEI** | 9 (18,0%) | 42 (42,4%) | **0,0028*** |
| **ARB** | 7 (14,0%) | 15 (15,2%) | 0,8528 |
| **Beta blockers** | 11 (22,0%) | 46 (46,5%) | **0,0035*** |
| **Betaagonist inhaled** | 4 (8,0%) | 16 (16,2%) | 0,1698 |
| **Glucocorticoids inhaled** | 3 (6,0%) | 5 (5,1%) | 0,8097 |
| **Vitamin D suplement** | 1 (2,0%) | 11 (11,1%) | 0,0541 |
| **Benzodiacepines** | 0 (0,0%) | 2 (2,0%) | 0,3149 |
| **Antidepressant** | 1 (2,0%) | 15 (15,2%) | **0,0142*** |
| **Ca-Antagonists** | 13 (26,0%) | 29 (29,3%) | 0,6756 |
| **Diuretics** | 10 (20,0%) | 42 (42,4%) | **0,0065*** |
| **Statins** | 6 (12,0%) | 37 (37,4%) | **0,0011*** |
| **Thyroxin substitution** | 6 (12,0%) | 17 (17,2%) | 0,4128 |
| **Antikonvulsives** | 3 (6,0%) | 10 (10,1%) | 0,4057 |
| **Antidiabetics** | 8 (16,0%) | 23 (23,2%) | 0,3077 |
| **Insulin** | 4 (8,0%) | 20 (20,2%) | 0,0563 |
| **Antibiotics** | 1 (2,0%) | 7 (7,1%) | 0,1973 |
| **Uricostatics** | 2 (4,0%) | 13 (13,1%) | 0,0813 |
| **PPI** | 7 (14,0%) | 46 (46,5%) | **0,0001*** |
| **Alpha antagonists** | 4 (8,0%) | 14 (14,1%) | 0,2805 |
| **NSARs except ASS** | 1 (2,0%) | 18 (18,2%) | **0,0050*** |
| **Opiats** | 1 (2,0%) | 16 (16,2%) | **0,0100*** |
| **Dopamin medication** | 2 (4,0%) | 4 (4,0%) | 0,9906 |
| **Vitamin supplements** | 3 (6,0%) | 16 (16,2%) | 0,0801 |
| **Antiarrhythmics** | 0 (0,0%) | 4 (4,0%) | 0,1517 |
| **Antihistaminics** | 1 (2,0%) | 15 (15,2%) | 0,0142 |

*ASS, acetylsalicylic acid; ACEI, Angiotensin-converting enzyme-inhibitors; ARB, angiotensin-II-blockers; NSAR, non-steroidal anti-inflammatory drugs*
