# Supplementary material for: Mortality rates of severe COVID-19-related respiratory failure with and without extracorporeal membrane oxygenation in the Middle Ruhr Region of Germany
Source: Sci Rep. 2023 Mar 29;13:5143. doi: 10.1038/s41598-023-31944-7 (PMC10054204; doi:10.1038/s41598-023-31944-7)
Supplement: Supplementary file 3 — Supplementary Information 3. [file 41598_2023_31944_MOESM3_ESM.docx]

***Supplementary Table 3:*** *Basic characteristics of studied patients. Comparison between waves through the pandemic.*

*First wave:1. February 2020 – 31. May 2020; Second wave: 1. June 2020 – 28. February 2021; 1. March 2021 – 31 July 2021*

| **Category** | **All patients 149 (100%)** | **First wave 15 (10,1%)** | **Second wave 96 (64,4%)** | **p value 1vs2** | **Third wave 38 (25,5%)** | **p value 1vs3** | **p value2vs3** |
| --- | --- | --- | --- | --- | --- | --- | --- |
| **Age, years** | 67 (24 - 90) | 68 (54 - 68) | 69 (24 - 90) | 0,8159 | 63 (36 - 86) | 0,1804 | 0,0160 |
| **Male** | 95 (63,7%) | 11 (73,3%) | 60 (62,5%) | 0,4210 | 24 (63,2%) | 0,4905 | 0,9439 |
| **ECMO-Therapy** | 50 (33,6%) | 2 (13,3%) | 30 (31,3%) | 0,1571 | 18 (47,4%%) | 0,0210 | 0,0805 |
| **Dead** | 108 (72,5%) | 6 (40,0%) | 73 (76,0%) | 0,0039 | 29 (76,3%) | 0,0113 | 0,9735 |
| **Comorbidities** | 146 (98%) | 15 (100%) | 93 (96,6%) | 0,4921 | 38 (100,0%) |  | 0,2738 |
| Hypertension | 107 (71,8%) | 12 (80,0%) | 69 (71,9%) | 0,5143 | 26 (68,4%) | 0,4090 | 0,6942 |
| Displemia | 49 (32,9%) | 3 (20,0%) | 36 (37,5%) | 0,1900 | 10 (26,3%) | 0,6380 | 0,2221 |
| DM | 54 (36,2%) | 3 (20,0%) | 37 (38,5%) | 0,1672 | 14 (36,8%) | 0,2449 | 0,8564 |
| Obesity | 95 (63,8%) | 8 (53,3%) | 61 (63,5%) | 0,4529 | 26 (68,4%) | 0,3114 | 0,5970 |
| Current smoker | 7 (4,7%) | 1 (6,7%) | 4 (4,2%) | 0,6676 | 2 (5,3%) | 0,8458 | 0,7840 |
| Renal insufficiency | 29 (19,5%) | 2 (13,3%) | 21 (21,9%) | 0,4524 | 6 (15,8%) | 0,8261 | 0,4324 |
| Allergies | 29 (15,4%) | 4 (26,7%) | 13 (13,5%) | 0,1926 | 6 (15,8%) | 0,3716 | 0,7390 |
| Heart disease | 56 (37,6%) | 6 (60,%) | 37 (38,5%) | 0,9151 | 13 (34,2%) | 0,6989 | 0,6433 |
| Atrial fibrillation | 26 (17,5%) | 4 (46,%) | 17 (17,7%) | 0,4146 | 5 (13,2%) | 0,2463 | 0,5252 |
| Cerebrovascular disease | 14 (9,4%) | 1 (6,7%) | 10 (10,4%) | 0,6548 | 3 (7,9%) | 0,8817 | 0,6595 |
| Autoimmun disease | 1 (0,7%) | 0 (0,0%) | 1 (1,0%) | 0,6945 | 0 (0,0%) |  | 0,5313 |
| Connective tissue disease | 1 (0,7%) | 0 (0,0%) | 1 (1,0%) | 0,6945 | 0 (0,0%) |  | 0,5313 |
| Liver disease | 3 (2%) | 0 (0,0%) | 3 (3,1%) | 0,4921 | 0 (0,0%) |  | 0,2738 |
| Any cancer | 15 (10,1%) | 0 (0,0%) | 11 (11,5%) | 0,1702 | 4 (10,5%) | 0,1984 | 0,8786 |
| Immunsupression condition | 4 (2,7%) | 0 (0,0%) | 3 (3,1%) | 0,4921 | 1 (2,6%) | 0,5350 | 0,8809 |
| Home oxygen therapy | 4 (2,7%) | 0 (0,0%) | 3 (3,1%) | 0,4921 | 1 (2,6%) | 0,5350 | 0,8809 |
| **previous medication** | 118 (79,2%) | 12 (10,%) | 77 (80,2%) | 0,9852 | 29 (76,3%) | 0,7780 | 0,6205 |
| Previous aspirin | 44 (29,5%) | 2 (13,3%) | 31 (32,3%) | 0,1377 | 11 (28,9%) | 0,2422 | 0,7094 |
| Antiplatelet | 7 (4,7%) | 0 (0,0%) | 6 (6,3%) | 0,3239 | 1 (2,6%) | 0,5350 | 0,4000 |
| Oral anticoagulant | 26 (17,4%) | 2 (13,3%) | 17 (17,7%) | 0,6790 | 7 (18,4%) | 0,6642 | 0,9235 |
| ACEI | 51 (34,2%) | 2 (13,3%) | 33 (34,4%) | 0,1047 | 16 (42,1%) | 0,0474 | 0,4061 |
| ARB | 22 (14,8%) | 2 (13,3%) | 16 (16,7%) | 0,7474 | 4 (10,5%) | 0,7766 | 0,3724 |
| Beta blockers | 57 (38,3%) | 3 (20,0%) | 45 (46,9%) | 0,0513 | 9 (23,7%) | 0,7780 | 0,0134 |
| Betaagonist inhaled | 20 (13,4%) | 1 (6,7%) | 15 (15,6%) | 0,3628 | 4 (10,5%) | 0,6722 | 0,4495 |
| Glucocorticoids inhaled | 8 (5,4%) | 0 (0,0%) | 6 (6,3%) | 0,3239 | 2 (5,3%) | 0,3747 | 0,8295 |
| Vitamin D suplement | 12 (8,1%) | 1 (6,7%) | 9 (9,4%) | 0,7362 | 2 (5,3%) | 0,8458 | 0,4383 |
| Benzodiacepines | 2 (1,3%) | 0 (0,0%) | 2 (2,1%) | 0,5768 | 0 (0,0%) |  | 0,3738 |
| Antidepressant | 16 (10,7%) | 1 (6,7%) | 13 (13,5%) | 0,4603 | 2 (5,3%) | 0,8458 | 0,1732 |
| Ca-Antagonists | 42 (28,2%) | 3 (20,0%) | 25 (26,0%) | 0,6201 | 14 (36,8%) | 0,2449 | 0,2178 |
| Diuretics | 52 (34,9%) | 4 (26,7%) | 39 (40,6%) | 0,3064 | 9 (23,7%) | 0,8243 | 0,0661 |
| Statins | 43 (28,9%) | 3 (20,0%) | 28 (29,2%) | 0,4663 | 12 (31,6%) | 0,4090 | 0,7852 |
| Thyroxin substitution | 23 (15,4%) | 3 (20,0%) | 14 (14,6%) | 0,5920 | 6 (15,8%) | 0,7194 | 0,8611 |
| Antikonvulsives | 13 (8,7%) | 3 (20,0%) | 7 (7,3%) | 0,1119 | 3 (7,9%) | 0,2178 | 0,9056 |
| Antidiabetics | 31 (20,8%) | 1 (6,7%) | 21 (21,9%) | 0,1724 | 9 (23,7%) | 0,1597 | 0,8225 |
| Insuline | 24 (16,1%) | 0 (0,0%) | 20 (20,8%) | 0,0515 | 4 (10,5%) | 0,1984 | 0,1632 |
| Antibiotics | 8 (5,4%) | 2 (13,3%) | 5 (5,2%) | 0,2324 | 1 (2,6%) | 0,1339 | 0,5192 |
| Uricostatics | 15 (10,1%) | 0 (0,0%) | 12 (12,5%) | 0,1498 | 3 (7,9%) | 0,2712 | 0,4498 |
| PPI | 53 (35,6%) | 6 (40,0%) | 35 (36,5%) | 0,7938 | 12 (31,6%) | 0,5686 | 0,5970 |
| Alpha antagonists | 18 (12,1%) | 1 (6,7%) | 13 (13,5%) | 0,4603 | 4 (10,5%) | 0,6722 | 0,6394 |
| NSARs except ASS | 19 (12,8%) | 2 (13,3%) | 15 (15,6%) | 0,8207 | 2 (5,3%) | 0,3258 | 0,1058 |
| Opiate | 17 (11,4%) | 1 (6,7%) | 15 (15,6%) | 0,3628 | 1 (2,6%) | 0,4969 | 0,0368 |
| Dopamin medication | 6 (4%) | 1 (6,7%) | 4 (4,1%) | 0,6676 | 1 (2,6%) | 0,4969 | 0,6754 |
| Vitamin supplements | 10 (12,8%) | 1 (6,7%) | 15 (15,6%) | 0,3628 | 3 (7,9%) | 0,8817 | 0,2401 |
| Antiarrhythmics | 4 (2,7%) | 1 (6,7%) | 3 (3,1%) | 0,4982 | 0 (0,0%) | 0,1122 | 0,2738 |
| Antihistaminics | 16 (10,7%) | 2 (13,3%) | 14 (14,6%) | 0,8991 | 0 (0,0%) | 0,0215 | 0,0126 |
| **Symptoms** |  |  |  |  |  |  |  |
| Dyspnea | 102 (68,5%) | 10 (66,7%) | 62 (64,6%) | 0,8765 | 30 (78,9%) | 0,3589 | 0,1078 |
| Tachypnea | 83 (55,7%) | 5 (33,3%) | 56 (58,3%) | 0,0714 | 22 (57,9%) | 0,1112 | 0,9633 |
| Fatigue | 63 (42,3%) | 6 (40,0%) | 38 (39,6%) | 0,9758 | 19 (50,0%) | 0,5205 | 0,2751 |
| Hipo-/Anosmia | 6 (4,0%) | 2 (13,3%) | 2 (2,1%) | 0,0298 | 2 (5,3%) | 0,3258 | 0,3333 |
| Disgeusia | 4 (2,7%) | 1 (6,7%) | 2 (2,1%) | 0,3130 | 1 (2,6%) | 0,4969 | 0,8481 |
| Sorethroat | 16 (10,7%) | 3 (20,0%) | 10 (10,4%) | 0,2873 | 3 (7,9%) | 0,2178 | 0,6595 |
| Fever | 63 (42,3%) | 8 (53,3%) | 39 (40,6%) | 0,3588 | 16 (42,1%) | 0,4691 | 0,8764 |
| Cough | 63 (42,3%) | 10 (66,7%) | 33 (34,4%) | 0,0168 | 20 (52,6%) | 0,3627 | 0,0519 |
| Vomiting | 11 (7,4%) | 0 (0,0%) | 9 (9,4%) | 0,2197 | 2 (5,3%) | 0,3747 | 0,4383 |
| Diarrhea | 16 (10,7%) | 2 (13,3%) | 11 (11,5%) | 0,8355 | 3 (7,9%) | 0,5507 | 0,5468 |
| Athromyalgia | 8 (5,4%) | 2 (13,3%) | 3 (3,1%) | 0,0775 | 3 (7,9%) | 0,5507 | 0,2320 |
| Synkope | 4 (2,7%) | 1 (6,7%) | 2 (2,1%) | 0,3130 | 1 (2,6%) | 0,4969 | 0,8481 |
| Chest pain | 5 (3,4%) | 1 (6,7%) | 2 (2,1%) | 0,3130 | 2 (5,3%) | 0,8458 | 0,3333 |
| Headache | 7 (4,7%) | 0 (0,0%) | 4 (4,2%) | 0,4253 | 3 (7,9%) | 0,2712 | 0,3858 |
| Rhinitis | 6 (4,0%) | 1 (6,7%) | 2 (2,1%) | 0,3130 | 3 (7,9%) | 0,8817 | 0,1113 |
| O2SAT<92% | 74 (49,7%) | 6 (40,0%) | 43 (44,8%) | 0,7311 | 25 (65,8%) | 0,0892 | 0,0285 |
| **Laboratory results at admission to ICU** |  |  |  |  |  |  |  |
| Lekocytes (/nl) |  | 7,79 ± 4,88 | 9,43 ± 4,88 | 0,2468 | 9,99 ± 4,94 | 0,1463 | 0,5677 |
| Lymphocytes (/nl) |  | 0,81 ± 0,56 | 0,88 ± 0,56 | 0,6958 | 0,91 ± 0,63 | 0,6429 | 0,8388 |
| Thrombocytes (/nl) |  | 177,92 ± 45,37 | 234,32 ± 123,8 | 0,1086 | 241,15 ± 99,57 | 0,0336 | 0,7750 |
| Hemoglobin (g/dl) |  | 12,02 ± 1,9 | 12,35 ± 2,45 | 0,6402 | 12,8 ± 2,37 | 0,2884 | 0,3548 |
| CRP (mg/dl) |  | 15,89 ± 11,79 | 19,58 ± 29,94 | 0,6627 | 14,74 ± 10,38 | 0,7428 | 0,3530 |
| Creatinin (mg/dl) |  | 1,21 ± 0,96 | 1,71 ± 1,99 | 0,3732 | 1,48 ± 1,69 | 0,5921 | 0,5377 |
| Bilirubin (mg/dl) |  | 0,59 ± 0,3 | 0,72 ± 0,83 | 0,5889 | 0,78 ± 0,84 | 0,4505 | 0,7311 |
| **Therapy** |  |  |  |  |  |  |  |
| Highflow_nasalcannula | 109 (73,2%) | 9 (60,0%) | 70 (72,9%) | 0,3087 | 30 (78,9%) | 0,1648 | 0,1648 |
| Non-invasive mechanical ventilation | 108 (72,5%) | 6 (40,0%) | 71 (74,0%) | 0,0077 | 31 (81,6%) | 0,0024 | 0,0024 |
| Invasive mechanical ventilation | 148 (99,3%) | 15 (100%) | 95 (99%) | 0,6945 | 38 (100,0%) |  |  |
| Tracheotomy | 48 (32,2%) | 4 (26,7%) | 31 (32,3%) | 0,6663 | 13 (34,2%) | 0,6044 | 0,6044 |
| Vasoactive treatment | 149 (100,0%) | 15 (100%) | 96 (100%) |  | 38 (100,0%) |  |  |
| Cytosorb | 36 (24,2%) | 2 (13,3%) | 19 (19,8%) | 0,5568 | 15 (39,5%) | 0,0684 | 0,0684 |
| **adjuvant therapy on ECMO or mechanical ventilation** | 144 (96,6%) | 15 (100%) | 93 (96,9%) | 0,4921 | 36 (94,7%) | 0,3747 | 0,3747 |
| neuromuscular blockage | 66 (44,3%) | 3 (20,0%) | 42 (43,8%) | 0,0828 | 21 (55,3%) | 0,0199 | 0,0199 |
| prone positioning | 117 (78,5%) | 14 (93,3%) | 75 (78,1%) | 0,1724 | 28 (73,7%) | 0,1164 | 0,1164 |
| nitrite oxide or prostacyclin | 1 (0,7%) | 0 (0,0%) | 1 (1,0%) | 0,6945 | 0 (0,0%) |  |  |
| Renal Replacement therapy | 81 (54,4%) | 8 (53,3%) | 50 (52,1%) | 0,9290 | 23 (60,5%) | 0,6399 | 0,6399 |
| **COVID19 treatment** |  |  |  |  |  |  |  |
| Use corticoids during admission | 23 (15,4%) | 0 (0,0%) | 20 (20,8%) | 0,0515 | 3 (7,9%) | 0,2712 | 0,2712 |
| Use corticoids during ICU | 135 (90,6%) | 4 (26,7%) | 93 (96,9%) | <0,0001 | 38 (100,0%) | <0,0001 | <0,0001 |
| Immunsupressants | 18 (12,1%) | 1 (6,7%) | 6 (6,3%) | 0,9513 | 11 (28,9%) | 0,0837 | 0,0837 |
| Antiviral drugs | 60 (40,3%) | 8 (53,3%) | 42 (43,8%) | 0,4923 | 10 (26,3%) | 0,0632 | 0,0632 |
| Remedesivir | 51 (34,2%) | 2 (13,3%) | 40 (41,7%) | 0,0356 | 9 (23,7%) | 0,4123 | 0,4123 |
| Tocilizumab | 15 (10,1%) | 1 (6,7%) | 4 (4,2%) | 0,6676 | 10 (26,3%) | 0,1164 | 0,1164 |
| Hydrocychloroquin | 9 (6,0%) | 8 (53,3%) | 0 (0,0%) | <0,0001 | 1 (2,6%) | <0,0001 | <0,0001 |

*ECMO, extracorproreal membrane oxygenation; SOFA, sepsis-related organ failure assessment score; ASS, acetylsalicic acid; ACEI, angiotensin-converting enzyme inhibitor; ARB, angiotensin-II-receptor blocker; Ca-Antagonist, Calcium-antagonist; PPI, proton-pump inhibitor; NSAR, non-steroidal antiinflammatory drugs; O2SAT, oxygen saturation at admission; CRP, c-reactive protein; ICU, intensive care unit*
