# Supplementary material for: Mortality rates of severe COVID-19-related respiratory failure with and without extracorporeal membrane oxygenation in the Middle Ruhr Region of Germany
Source: Sci Rep. 2023 Mar 29;13:5143. doi: 10.1038/s41598-023-31944-7 (PMC10054204; doi:10.1038/s41598-023-31944-7)
Supplement: Supplementary file 4 — Supplementary Information 4. [file 41598_2023_31944_MOESM4_ESM.docx]

***Supplementary Table 4:*** *Bleeding complications. Comparison between ECMO survivors and ECMO non-survivors*

| **Category** | **ECMO non-survivors 40/50 (80%)** | **ECMO survivors 10/50 (20%)** | **p value** |
| --- | --- | --- | --- |
| Any relevant bleeding | 27 (67,5%) | 7 (70%) | 0,8825 |
| Nasopharyngeal | 15 (37,5%) | 3 (30%) | 0,6663 |
| Brain | 5 (12,5%) | 1 (10%) | 0,8320 |
| Injection site (ECMO or drainages) | 9 (22,5%) | 1 (10%) | 0,3871 |
| Gastrointestinal | 6 (15%) | 1 (10%) | 0,6909 |
| Lung | 2 (5%) | 2 (20%) | 0,1227 |
| Heart | 1 (2,5%) | 0 (0%) | 0,6221 |

*ECMO, extracorproreal membrane oxygenation*
